# P03-015 - Dapson treats chronic Pupura Schoenlein (PSH)

**DOI:** 10.1186/1546-0096-11-S1-A213

**Published:** 2013-11-08

**Authors:** G Dueckers, M Frosch, C Assaf, H Wiggermann, D Schneider, T Niehues

**Affiliations:** 1 Childrens Hospital, Helios Kliniken, Krefeld, Germany; 2 Pediatrics, Vestische Kinderklinik, Datteln, Germany; 3 Dept. Dermatology, Helios Klinken, Krefeld, Germany; 4 Pediatrics, Private Practice, Schwerte, Germany; 5 Childrens Hospital, Kliniken Dortmund GmbH, Dortmund, Germany

## Introduction

PSH is common and, with few exceptions, resolves spontaneously. In complicated cases treatment can be difficult.

## Case Report

Here we present an 11 year old Iranian girl, of healthy parents suffered from chronic PSH with chronic exanthema, severe ulcerative dermatitis and arthralgia. Clinical observation: no fever, no arthritis, no signs of polyserositis or hepatosplenomegaly; Laboratory: normal values (full blood count, CRP, complement (C3, C4), no autoantibodies (ANA, ANCA, anti-ds-DNA), no cryoglobulins, zinc, IgG, IgM). Abnormal findings: highly elevated IgA in serum (maximum 2030 mg/dl), elevation of ESR and MRP8/ 14. Skin biopsy: leukocytoclastic infiltrates in subcutaneous fatty tissue. Immunohistochemistry: IgA deposits in tip of papillae and upper corium. No mutation in Marenostrin was found. Multimodal therapeutic approaches (Cortisone, Methotrexate, Azathioprine, Colchicine, i.v. Immunoglobulins) remained without success for 8 years. With the administration of Dapsone symptoms resolved within days and remain under control for > 8 months now. Met-Hb level is tolerable.

## Discussion

Anti-inflammatory potency of dapsone is illustrated. Therapeutic efficacy of Dapsone has been reported in chronic PSH[1,2], but the mechanism remains to be fully elucidated. Hypothesis: Endothelial cells and hyperreactive B-cells (as illustrated by IgA elevation) secrete IL-8. IL-8 is elevated in patients with PSH [3]. IL-8 stimulates perivascular invasion by neutrophils. Dapsone can inhibit secretion of IL-8 [4], thereby impairing neutrophil function [5-7].

In conclusion Dapsone might be benefical for complicated cases of PSH. The mechanism of its anti-inflammatory potency remains to be elucidated.

## Competing interests

None Declared.
